# Spindly leg syndrome in *Atelopus varius* is linked to environmental calcium and phosphate availability

**DOI:** 10.1371/journal.pone.0235285

**Published:** 2020-06-29

**Authors:** Elliot Lassiter, Orlando Garcés, Kathleen Higgins, Eric Baitchman, Matthew Evans, Jorge Guerrel, Eric Klaphake, Donna Snellgrove, Roberto Ibáñez, Brian Gratwicke

**Affiliations:** 1 Panama Amphibian Rescue and Conservation Project, Smithsonian Tropical Research Institute, Panama City, Panamá; 2 Simon Fraser University, Burnaby, British Columbia, Canada; 3 Zoo New England, Boston, MA, United States of America; 4 Reptile Discovery Center, Smithsonian’s National Zoological Park, Washington, DC, United States of America; 5 Cheyenne Mountain Zoo, Colorado Springs, Colorado, United States of America; 6 Waltham Petcare Science Institute, Waltham on the Wolds, Leicestershire, England, United Kingdom; 7 Sistema Nacional de Investigación, Secretaría Nacional de Ciencia, Tecnología e Innovación, Panama City, Panamá; 8 Center for Species Survival, Smithsonian National Zoo and Conservation Biology Institute, Front Royal, Virginia, United States of America; Universitat Trier, GERMANY

## Abstract

Spindly leg syndrome (SLS) is a relatively common musculoskeletal abnormality associated with captive-rearing of amphibians with aquatic larvae. We conducted an experiment to investigate the role of environmental calcium and phosphate in causing SLS in tadpoles. Our 600-tadpole experiment used a fully-factorial design, rearing *Atelopus varius* tadpoles in water with either high (80mg/l CaCO_3_), medium (50mg/l CaCO_3_), or low calcium hardness (20mg/l CaCO_3_), each was combined with high (1.74 mg/l PO_4_) or low (0.36 mg/l PO_4_) phosphate levels. We found that calcium supplementation significantly improved tadpole survival from 19% to 49% and that low calcium treatments had 60% SLS that was reduced to about 15% at the medium and high calcium treatments. Phosphate supplementation significantly reduced SLS prevalence in low calcium treatments. This experimental research clearly links SLS to the calcium: phosphate homeostatic system, but we were unable to completely eliminate the issue, suggesting an interactive role of other unidentified factors.

## Introduction

Spindly leg syndrome (SLS) is a musculoskeletal abnormality commonly associated with captive-rearing of amphibian aquatic larvae, resulting in underdeveloped limbs that cannot support the body of newly metamorphosed animals [1–5]. Animals have malformed joints and reduced numbers of shortened, thinner muscle fibers in the limbs, some of which are detached [6]. The issue has been widely discussed in the amphibian husbandry community as a limiting factor for captive-breeding efforts of frogs with aquatic larvae. While there are many speculated causes of SLS, there have been few adequately replicated studies on the topic. Nutritional deficiencies in the diet have been most widely postulated as the causative factor in SLS, but one recent study found that SLS was prevalent, even when feeding presumed nutritionally-complete diets [4].

Four independent studies suggested that soft water was related to skeletal deformities in tadpoles and metamorphs [4,6–8]. Calcium is an important mineral for tadpoles because as they metamorphose, their cartilage skeletons begin to ossify, creating a high demand for calcium [9]. The calcium is stored in endolymphatic sacs and used in metamorphosis, and the Ca plasma concentrations in larval amphibians are substantially lower than adults [9–11]. Tadpoles reared in deionized water experience poor neurological development leading to limb malformation [7], but addition of calcium improves survival [6], reduces larval deformities [8], and reduces the incidence of malformations in the limbs [7]. A previous study by our own research group found that SLS incidence was aggravated by overfeeding tadpoles, and ameliorated by filtering the water through a reverse-osmosis (RO) membrane and then reconstituting it [4]. We hypothesized that soft tap water at our facility may have led to calcium being a limiting factor and that adding calcium chloride to RO water may have reduced SLS prevalence. We also hypothesized that phosphates released from uneaten food may have altered the Ca:P ratio of the water in the overfed treatments, further aggravating the problem [4,5].

Serum calcium and phosphate concentrations are homeostatically regulated through intestinal absorption, bone mineral deposition, and kidney mineral excretion (DiMeglio and Imel, 2019). When serum concentrations of Ca are low, parathyroid hormone increases intestinal absorption, bone and renal resorption (Blaine et al., 2015; DiMeglio and Imel, 2019). Inadequate Ca:P ratios in diets or factors affecting the regulation of calcium metabolism such as insufficient UVb exposure and vitamin D_3_ deficiencies have been associated with symptoms of a metabolic bone disease in adult amphibians [9,12–14]. While dietary Ca:P ratios of 1:1–1.2 are recommended for feeding adult reptiles and amphibians [14], it is unclear whether high phosphate concentrations in the water affects calcium metabolism in tadpoles. Experimental evaluations of phosphate toxicity found no effect on tadpole survival, growth or development [15], but there is a perception among amphibian husbandry professionals that excessive phosphates are undesirable for tadpole development [16].

We conducted a controlled, laboratory-rearing experiment to determine the effects of calcium and phosphate concentrations on the prevalence of SLS in *Atelopus* tadpoles. We hypothesized that SLS is induced by insufficient bodily stores of calcium for metamorphosis, or an imbalance in calcium: phosphate ratios in the water.

## Methods

### Experimental design

We used a fully-factorial design with high, medium, and low calcium, each replicated with high or low phosphate levels for a total of six treatment groups. Each treatment was replicated in five tanks with 20 tadpoles per tank for a total of 30 tanks and 600 *Atelopus varius* tadpoles. Water for each treatment was prepared by filtering tap water (through an AquaFX 100 GPD reverse osmosis membrane) that removed 80–90% of the total dissolved solids. The RO water was reconstituted using 46.5 mg MgSO_4_ 35.8 mg KHCO_3_, and 29.8 mg NaHCO_3_ per liter following Association of Zoos and Aquariums (AZA) RO reconstitution recipe [17]. Calcium treatments were prepared with 0 (low), 39.5 (medium), or 79 (high) mg CaCl_2_ per liter of reconstituted water. The same medium calcium concentrations are recommended in the AZA recipe used by Camperio Ciani et. al. [4], and we doubled that concentration for the high treatment. The phosphate treatments were obtained by adding either 0 or 2.71 mg Na_3_PO_4_ per liter of water for the low and high treatments respectively. We selected a quantity of sodium orthophosphate that would increase the phosphorous (P) concentration in the RO water by 0.5mg/l. About 87% of natural streams in the US have phosphorous (P) concentrations lower than 0.5mg/l, which is considered a high concentration in streams [18]. As reference to natural conditions, calcium hardness and phosphate levels were measured in 2 streams with healthy populations of *Atelopus varius*; which were determined to be classified as “soft” (14.2– 18mg/l hardness) and low in phosphates (0–0.07 mg/l) (S1 Data, *Atelopus* Stream Water Quality). This design received approval from the Smithsonian Tropical Research Institute’s Animal Care and Use Committee 2018-0427-2021.

### Husbandry

A clutch of *Atelopus varius* eggs was laid at the Panama Amphibian Rescue and Conservation Project’s Gamboa facility on Jan 10, 2019 and reared in that tank containing carbon-filtered tap water for 27 days until Feb 6, 2019. We then collected 600 *Atelopus varius* tadpoles from the same clutch of eggs and divided into thirty randomly selected groups of 20 tadpoles at Gosner stage 20–25 [19] before placing them in the 20-litre aquariums 40cm long x 20 cm wide x 25 cm high without any gravel or lighting other than ambient fluorescent ceiling lights. Water was filtered using a small Hikari Aquarium Solutions Bacto-Surge Foam Filter with an airstone in each tank plus one supplemental air stone to improve circulation. Water was partially (30%) changed three times per week through siphoning. Each tank was fed 0.125g of a nutritionally-balanced food developed at the Waltham Center for Pet Nutrition that contained 1.8% calcium (Table 1). The food was weighed then mixed with water to make a paste that was spread to ~1mm thickness on an acrylic plate and dried as a film. Each day the feeding plates were replaced in each tank. Once per month, immediately prior to water changes, CaCO_3_ and PO_4_ levels were measured using HANNA HI720 and HI713 test kits. For all tanks, the mean temperature was 20.9°C +/- 0.7 °C SD (stream temperatures at sites where parents were collected ranged from 22–26°C RI, unpublished data); the mean pH 7.5 +/- 0.2 SD; the mean dissolved oxygen 89.7% +/- 3.7% SD; the mean NH_4_ 2.1 mg/l +/- 1.1 mg/l SD; and NH_3_ was not detected (S1 Data, Water Quality).

**Table 1:** Nutritional analysis of tadpole food (% dry matter basis) batch prepared by the Waltham Petcare Science Institute.

Analysis		Unit
Proximates		
Ash	11.7	%
Crude Fibre	1.8	%
Fat	7.5	%
Moisture	3.2	%
Protein	38.5	%
Vitamins		
Vitamin B_1_	28.8	mg/kg
Vitamin B_2_	41.1	mg/kg
Vitamin D_3_	4790	IU/kg
Vitamin A	30400	IU/kg
Vitamin E	510	mg/kg
Pantothenic acid	163	mg/kg
K3	15.9	mg/kg
Niacin (B_3_)	391	mg/kg
Choline	2910	mg/kg
B_6_	28.9	mg/kg
B_12_	0.084	mg/kg
Folic acid	14.4	mg/kg
Vitamin C	2220	mg/kg
Minerals		
Copper	20.3	mg/kg
Calcium	1.82	%
Magnesium	0.187	%
Manganese	73.7	mg/kg
Iron	723	mg/kg
Potassium	0.764	%
Sodium	0.772	%
Zinc	96.7	mg/kg
Phosphorus	1.12	%
Essential Fatty Acids		
Linoleic	0.578	%
Linolenic	0.087	%
Arachidonic	0.055	%
Eicosapentaenoic	0.343	%
Docosahexaenoic	0.301	%
Amino Acids		
Aspartic acid	3.15	%
Serine	1.54	%
Glutamic Acid	6.15	%
Glycine	2.4	%
Histidine	0.8	%
Arginine	2.19	%
Threonine	1.43	%
Alanine	2.05	%
Proline	2.14	%
Tyrosine	1.12	%
Valine	1.63	%
Methionine - Total Analysis	1	%
Lysine	2.49	%
Isoleucine	1.4	%
Leucine	2.53	%
Phenylalanine	1.5	%
Cysteine	0.37	%

When tadpoles developed hind limbs, floating polystyrene slices were placed in the tanks to facilitate froglet emergence. For each tank we recorded 1) prevalence of SLS by classifying each metamorph into ‘spindly’ or ‘healthy’. All SLS frogs exhibited in SLS in the fore-limbs only (Claunch and Augustine, 2015, Fig 1); 2) mortality or survival through metamorphosis Gosner Stage 46, [19]; and 3) snout-vent-length (SVL) and weights of metamorphs. Any animals with SLS were euthanized by 20% benzocaine gel applied directly to the skin as advocated in AVMA humane euthanasia guidelines (AVMA, 2007).

**Fig 1:**
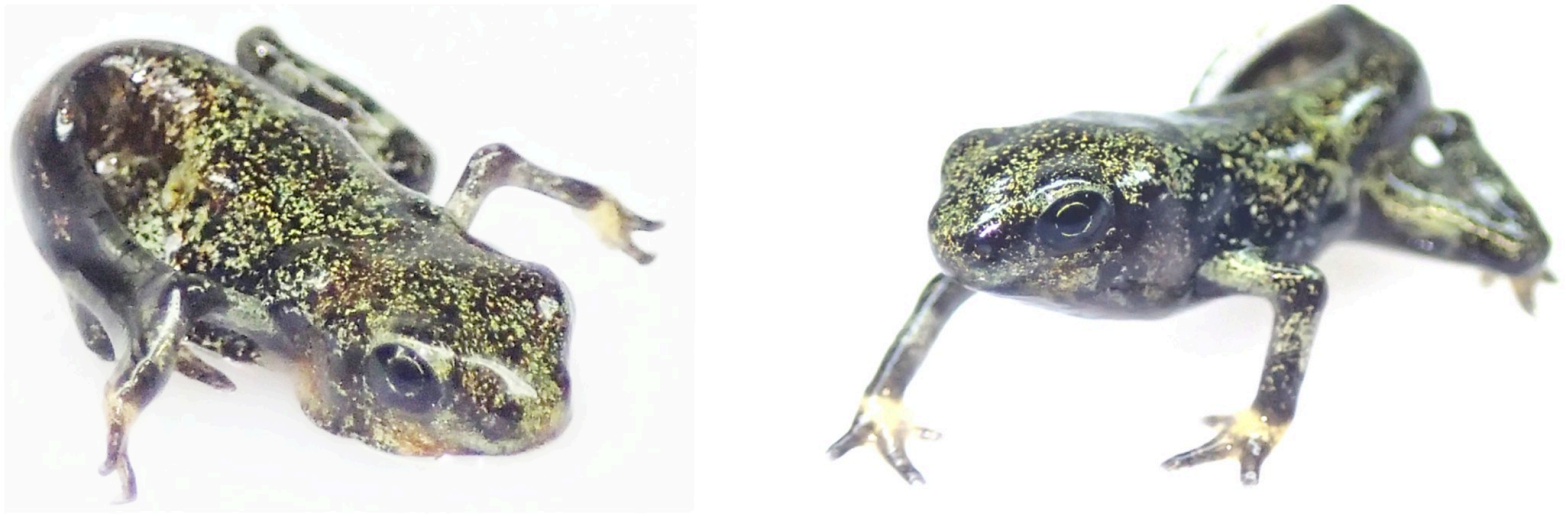
*Atelopus varius* metamorphs: Typical spindly leg syndrome posture (left) and normal, healthy posture (right).

### Statistical analysis

A previous study demonstrated that this sample size was sufficient to highlight a 10% difference in survival between groups with a power of 80% [4]. Data were visualized by plotting mean values for each tank, which served as the experimental unit, with corresponding standard error bars. We examined data for assumptions of normality and homogeneity of variance by examining residual plots, difference showing P<0.05 were considered significant. Only animals reaching metamorphosis are included in the statistical analysis. The independent effects of calcium and phosphate, and their interaction were modeled using a linear model in R with the package ‘car’ testing the experimental treatment effects on the following response variables; 1) SLS prevalence in metamorphs; 2) tadpole survival to metamorphosis; and 3) size of metamorph measured by SVL and weight. Model = lm(“responsevariable” ~ phosphate + calcium + phosphate: calcium, data = data), results were reported as a type II ANOVA [20]. We used survdiff in the R package survival for R [21] to conduct a log-rank test [22] for differences in survivorship between the 6 treatment groups.

## Results

**Fig 2:**
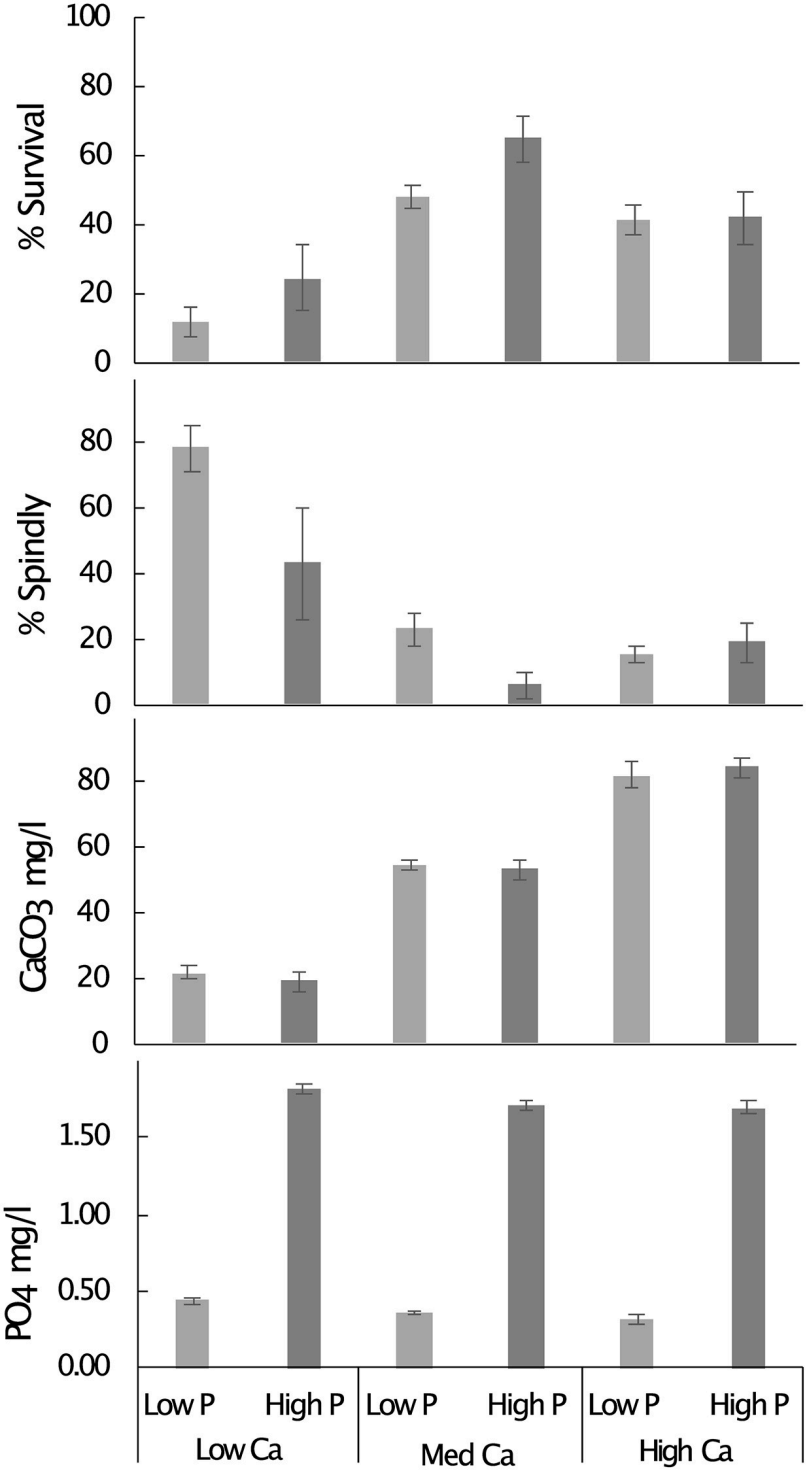
Effects of phosphate and calcium on survival to metamorphosis and prevalence of spindly leg syndrome among surviving *Atelopus varius* metamorphs. Shown are means +/- SE (bars). Each treatment was replicated in 5 tanks of 20 tadpoles, CaCO_3_ and PO_4_ was measured monthly in each tank for 4 months.

**Fig 3:**
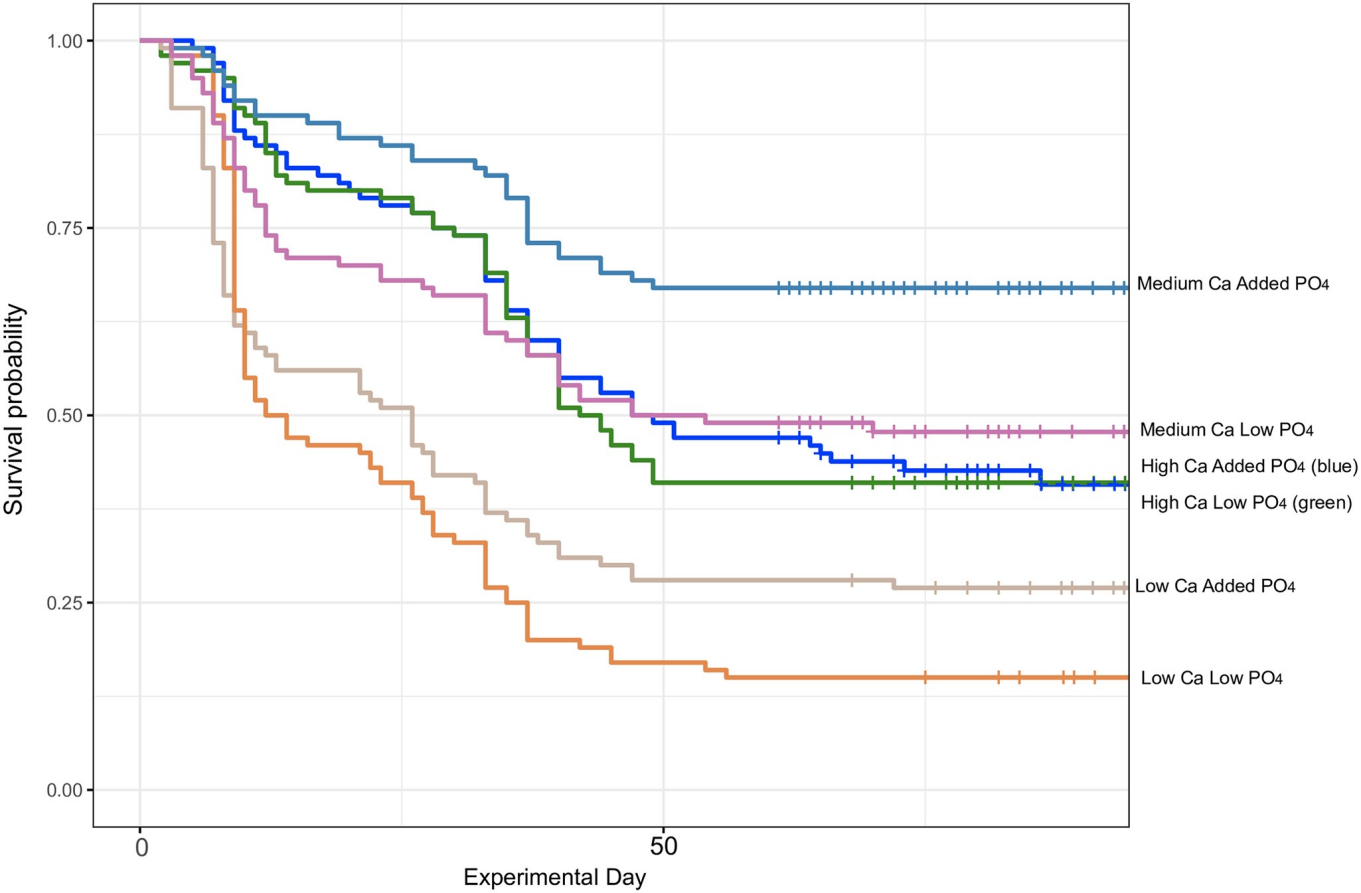
Survival probability curves for the 6 experimental treatment groups, cross hatches indicate censored animal(s) that metamorphosed and were censored from this survivorship analysis at that point. A log-rank test (Table 3) revealed highly significant differences between groups, Chisq = 94 on 5 degrees of freedom, p = <2e-16.

The addition of calcium to create a mean calcium hardness of ≥50mg/l markedly improved *Atelopus varius* tadpole survival to metamorphosis (P < 0.0001) (Fig 2, Table 2). Overall, the four treatments with added calcium experienced 49% survivorship, but this was reduced to 19% in the treatment groups with no additional calcium which had a mean calcium hardness of about 20mg/l (Fig 2). The addition of phosphate had no significant effect on survival (P = 0.1461, Table 1), and there was no significant interaction between Ca and P. A survivorship analysis indicated that the largest differences from expected values were in the medium Ca, added PO4 treatments and the two low Ca treatments (Fig 3, Table 3).

**Table 2:** 2-way ANOVA testing for the experimental effects of added calcium and phosphate on *Atelopus varius* tadpole survival, and prevalence of spindly leg syndrome in *A*. *varius* metamorphs.

**% Survival**				
	Sum of Squares	DF	F Value	Probability
Phosphate	0.0507	1	2.263	0.1461
Calcium	0.6912	2	5.406	0.00006 ***
PhosphateCalcium	0.0326	2	0.726	0.4942
**% Spindly Leg Syndrome**				
	Sum of Squares	DF	F Value	Probability
Phosphate	0.1729	1	4.817	0.038 *
Calcium	1.2135	2	16.90	0.00003 ***
Phosphate*Calcium	0.1739	2	2.422	0.111

**Table 3:** Log-Rank test for differences in survival probability between the 6 treatment groups. Chisq = 94 on 5 degrees of freedom, p = <2e-16.

	N	Observed	Expected	(O-E)^2/V	(O-E)^2/E
High Ca Added PO4	100	58	68.4	1.59	2.04
High Ca Low PO4	100	59	67	0.964	1.23
Low Ca Added PO4	100	75	46.5	17.417	20.79
Low Ca Low PO4	100	88	44.8	41.69	51.75
Medium Ca Added PO4	100	34	75.5	22.82	30.13
Medium Ca Low PO4	100	52	63.7	2.148	2.71

Adding calcium was associated with a highly significant reduction in SLS prevalence from about 60% without supplemented calcium to 15% in high and medium calcium-supplemented treatments (P < 0.0001, Fig 2, Table 1). As with survival, it appears that once a threshold of 50mg/l CaCO_3_ was reached, additional calcium did not further reduce the incidence of SLS. The addition of phosphate to the water also reduced SLS prevalence in the low-calcium treatment (P <0.05, Fig 2, Table 1). Overall, SLS prevalence averaged 23% in groups with added phosphate and 39% in groups with no added phosphate.

There was no significant effect of any treatment on the size or weight of froglets at metamorphosis which took 105 days +/- 15 days (SD). *Atelopus varius* size at emergence measured 8 mm SVL +/- 0.6mm SE, and weighed 0.086 g +/- 0.06g SE and SLS metamorphs did not significantly differ from healthy metamorphs in weight, length, or number of days to metamorphosis (two sample t test, P > 0.05, S1 Data, Experimental Data).

## Discussion

Even though the diet offered to tadpoles contained 1.8% calcium, we found lower tadpole survival and increased prevalence of SLS in water with calcium hardness lower than 50mg/l. Tadpoles absorb calcium from their environment primarily across the gills (70%) and skin (25%) [23], thus the supplementation of calcium to the water is likely more effective for tadpoles rather than by diet. Others have reported that limitation of calcium in the rearing water is directly correlated with the accumulation of calcium in endolymphatic sacs and skeletal mineralization [11]. Our observed threshold for calcium hardness as a limiting factor was between 20–50mg/l CaCO_3_ for *Atelopus varius*. Water hardness of the tap water in the facility fluctuated from 5–50 mg/l (S1 Data, Source Water Quality), levels classified by the United States Geological Survey as “soft”. This may explain some of the observed variation in SLS prevalence over time. Supplementation of filtered tap water with CaCl_2_ was more cost-effective than preparing fully reconstituted RO water. It is unclear whether the observed CaCO_3_ threshold will apply to other taxa or situations. Haakvoort et al. [6]. reported 100% cases of SLS in dart frogs at 100, 10 and 1 mg/l CaCO_3_.

Water supplementation of phosphate was associated with a significant reduction of SLS. The experimental effect, however, was less pronounced than the supplementation of calcium (Fig 2, Table 2), and phosphate supplementation did not significantly affect survivorship. This finding provides further evidence that SLS, at least in *Atelopus varius*, is connected to an imbalance in calcium and phosphate homeostasis, but it suggests that the hypothesis that high phosphate levels and high calcium: phosphorous ratios in the water are the not the cause of SLS in this species.

In this experiment, even the unsupplemented group’s water would fall within the ranges of those parameters in the two natural reference streams. Experimentally, calcium water supplementation accounted for most of the observed variation in survival and SLS prevalence, but there still was 10–20% SLS in calcium-supplemented treatments; suggesting a potential role of some other factor, such as overfeeding [4], which was untested in this experimental design. Alternatively, SLS may be a more common condition in nature than is currently appreciated, there is at least one anecdotal report of SLS in the wild *Ranitomeya ventrimaculata* from Río Napo/ Ecuador (pers. comm. H. Divossen).

All tadpoles were fed the same quantity of food, but we have not yet determined a feeding regime where food quantity limits growth. It is possible, therefore, that all tadpoles in this experiment were overfed as uneaten food remained on food plates at each change of food plates. Growth and differentiation may be partially decoupled from each other in poikilotherms through genetic modification or through rapid growth associated with high food availability [24]. For example, growth-enhanced transgenic salmon are known to be inferior swimmers with muscle hyperplasia and reduced muscle fiber size [25,26]. Faster growth rates are associated with reduced burst swimming speed in tadpoles [27], shorter legs in froglets [28], and food-limited tadpoles have improved bone ossification in froglets [29]. One potential explanatory hypothesis tying these observations together is that rapid growth rates through calories absorbed in the intestinal tract could become decoupled from rates of normal sequestration of calcium across the gills, that may manifest as SLS, particularly in low calcium, low phosphate environments. The fact that we only observed SLS in the forelimbs, which are the last to emerge, may be indicative of depletion of reserves that were available to the tadpoles earlier in metamorphosis.

In conclusion, our management recommendations for husbandry and veterinary professionals seeking to reduce or eliminate SLS in anurans are to 1) test water hardness and consider supplementing water softer than 50 mg/l calcium hardness with CaCl_2_ (this study) and 2) to avoid overfeeding tadpoles which was associated with increased SLS prevalence in an earlier study [4]. Future SLS studies may benefit by incorporating calorie limitation or examining other potentially synergistic factors that may decouple growth and development in calcium-limited environments to obtain further insights into the mechanisms responsible for SLS. A detailed comparison of husbandry and feeding protocols and water chemistry with other *Atelopus* rearing programs that have had no SLS may help to identify alternate factors.

## Supporting information

S1 Data (XLSX)
